# Neuropsychiatric Comorbidity in Primary Hyperparathyroidism Before and After Parathyroidectomy: A Population Study

**DOI:** 10.1007/s00268-022-06485-1

**Published:** 2022-03-05

**Authors:** A. Koman, R. Bränström, Y. Pernow, R. Bränström, I.-L. Nilsson, Fredrik Granath

**Affiliations:** 1 Department of Breast, Endocrine Tumors and Sarcoma, Karolinska University Hospital, Stockholm, Sweden; 2 Department of Molecular Medicine and Surgery, Karolinska Institutet, 171 76 Stockholm, Sweden; 3 Department of Endocrinology, Karolinska University Hospital, Stockholm, Sweden; 4 Department of Clinical Neuroscience, Karolinska Institutet, Stockholm, Sweden; 5 Department of Medicine Solna, Division of Clinical Epidemiology, Karolinska Institutet, Stockholm, Sweden

## Abstract

**Background:**

Primary hyperparathyroidism (PHPT) is often accompanied by neuropsychiatric symptoms. This study aimed to map out psychiatric comorbidity as reflected by medical treatment for psychiatric symptoms.

**Methods:**

A retrospective case–control analysis and a prospective cohort analysis of psychotropic drug utilization before and after PTX. A total of 8279 PHPT patients treated with parathyroidectomy in Sweden between July 1, 2008 and December 31, 2017 compared to a matched control cohort from the total population (*n* = 82,790). Information on filled prescriptions was collected from the Swedish Prescribed Drug Register (SDR). Socioeconomic data and diagnoses were added by linkage to national patient and population registers. Regression analyses were used to calculate relative drug utilization (OR) within 3 years prior to PTX and relative incidence of drug treatment (RR) within 3 years postoperatively.

**Results:**

Utilization of antidepressant, anxiolytic and sleep medication was more comprehensive in PHPT patients compared with the controls prior to PTX. The most common were benzodiazepines [OR 1.40 (95% CI: 1.31–1.50)] and selective serotonin reuptake inhibitors [SSRI; OR 1.38 (95% CI: 1.30–1.47)]. Postoperatively, the excess prescription rate for anxiolytic benzodiazepines decreased within three years from a 30 to 19% excess and for benzodiazepines for sleep from 31 to 14%. No corresponding decrease in excess prescription rate was observed for SSRI.

**Conclusion:**

PHPT is associated with increased utilization of antidepressive medications and benzodiazepines before PTX. This study implies that psychiatric symptoms should be considered in PHPT patients and continuous medication should be reevaluated after PTX.

**Supplementary Information:**

The online version contains supplementary material available at 10.1007/s00268-022-06485-1.

## Introduction

Today, primary hyperparathyroidism (PHPT) is a common disease that ranks third among endocrine diagnoses [1]. PHPT is often accompanied by a wide array of nonspecific neuropsychiatric symptoms which are often indistinguishable from symptoms related to other conditions. Significant neuropsychiatric symptoms, namely depression, anxiety, cognitive decline, fatigue and sleep disorders may be present even in a biochemically mild disease where an indisputable indication for surgical treatment is lacking. Positive effects on the overall quality of life (QoL) and cognitive function commonly occurs after PTX [1–3]. The surgical procedure is safe and effective [4–8]. Though the occurrence of neuropsychiatric symptoms related to PHPT is not controversial today, the clinical significance and the pathophysiology is debated [2, 9–12]. Still, it remains challenging to predict if the patient will benefit from PTX [13–15].

Previous knowledge was mainly based on self-reported QoL data and it is thus unknown whether healthcare and drug consumption related to neuropsychiatric comorbidity in untreated PHPT had an impact at a population level. The hypothesis behind this study was that treatment for neuropsychiatric morbidity is more prevalent in patients with PHPT compared with the background population. The specific aim was to investigate the preoperative utilization of psychotropic drugs within three years prior to PTX in comparison with the background population. Second to that, the aim was to analyze any potential implications on psychiatric comorbidity after PTX.

## Methods and material

### Study population

**Fig. 1:**
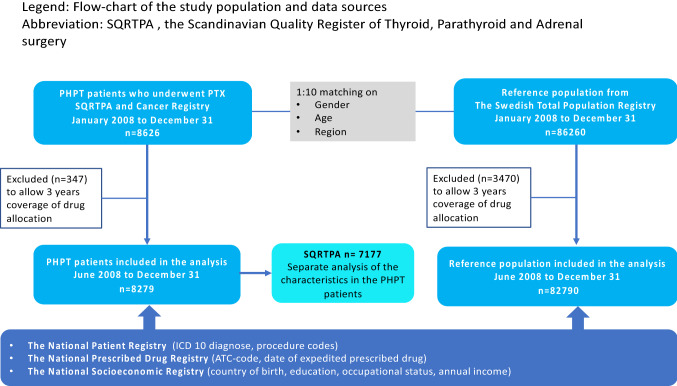
Flow-chart of the study population and data sources. *SQRTPA* the Scandinavian Quality Register of Thyroid, Parathyroid and Adrenal surgery

All patients (*n* = 8626) registered with PTX between January 1, 2008 to December 31, 2017 in the Scandinavian Quality Register of Thyroid, Parathyroid and Adrenal surgery (SQRTPA) [16] as well as in the Cancer Registers, were collected by the National Board of Health and Welfare [17]. For each case, Statistics Sweden (SCB) selected 10 individuals from the Total Population Register matched by year of birth, gender and county (*n* = 86,260) [18]. To allow complete analysis of drug allocation within 3 years before PTX, 347 patients registered between January and June 2008 and their respective controls (*n* = 3470) were excluded from analyses resulting in a study population of 8279 PHPT and 82,790 controls (Fig. 1).

### Data sources

The SQRTPA was founded in 2004 and has a well-validated database that covers about 95% of all PTX performed in Sweden [16]. SQRTPA comprises detailed information on any underlying causes (e.g., renal failure, multiple endocrine neoplasia 1, lithium) biochemical measures, symptoms, indications for surgery, postoperative complications and pathology reports from the time of referral up until 6 months postoperatively.

PTX patients were identified in SQRTPA and/or the Swedish Cancer Register [17]. Population control subjects were sampled as non-PTX subjects and matched according to patients’ sex, age and county as listed in The Swedish Total Population Registry [18]. Information on specialist care visits and hospital admissions was retrieved from The National Patient Registry, and information on income, education level and country of origin was collected from Longitudinal Integrated Database for Health Insurance and Labor Market Studies [19, 20]. Information on administered prescribed drugs was retrieved from the Swedish Prescribed Drug Register [21] containing continuous information on all filled prescriptions in Sweden. Information on vital status and emigration was collected from RTB [18].

### Study design

The index date was defined as the time of PTX for patients and the same date for the matched population controls. The primary outcome measure was the utilization of psychotropic drugs. Second to that, a selection of diagnoses (ICD-10) which were deemed relevant for the research question (e.g., cognitive and psychiatric diagnoses, gastrointestinal, kidney stone and chronic musculoskeletal diseases) was mapped for both cohorts during one and five years prior to index date. Socioeconomic status, educational level and household income the year before the index date were considered as confounders in addition to the matching variables. The retrospective case–control study comprehended drugs according to ATC codes designated to match the treatment of neuropsychiatric disorders. Data on prescriptions filled and administered by a pharmacy) during a period of 3 years before and after index date were retrieved, which was considered relevant to detect any change of clinical significance. The retrospective case–control study comprehended drugs according to ATC codes designated to match the treatment of neuropsychiatric disorders and cognitive deficiency. The prevalence of utilization within one and three years before index date was assessed and compared between patients and controls. Interactions in relation to calcium levels, age and gender of the patient were analyzed. In the prospective cohort study, the study subjects were divided into incident and prevalent users based on whether they filled at least one prescription during the year before the index date. End of follow-up was defined as date of death, date of emigration or December 31 2019, whichever occurred first. Administrations of psychotropic drugs three years after the index dates were compared by calculating the incidence of at least one administered filled prescription per six months period.

Power calculations were based on the assumption of a Poisson. The population was estimated to guarantee 80% statistical power at a 95% confidence level for the presence of risk factors between 0.5–30% among controls in the retrospective case–control analyses and an incidence 0.5–10% among controls in the prognostic cohort study.

Base-line characteristics are presented as median and interquartile range for continuous variables and as proportions for categorical variables. The cohorts were stratified into age groups: <50 years, 50–64 years, 65–79 and >80 years.

Drug utilization (filled prescriptions) preoperatively was compared by conditional logistic regression, and results are presented as odds ratios (OR) together with 95% confidence intervals (95% CI). Effect modification by calcium levels, in quartiles, was assessed by an appropriate interaction term. Analyses of the prescription rates after PTX in 6 months periods in patients and controls were analyzed with repeated measurement Poisson regression models. Comparisons were adjusted for age, gender, region, income, ethnicity and education. Trends in the RRs over time were tested by including time as a continuous variable. The results are presented as prescription rates and as rate ratios (RR) with 95% CI. *p* values less than 0.05 were considered significant. Analyses were performed using SAS 9.4 (SAS Institute Inc., Cary, NC, USA) and SPSS version 27.

## Results

Baseline characteristics at index date, including age distribution, information on education, employment, and country of birth are presented in Table 1. Clinical characteristics of the 7177 individuals registered in SQRTPA are presented in Table 2. Among patients with data available for calcium levels, (*n* = 6932), 95.4% remained normocalcemic six months postoperatively.

**Table 1:** Characteristics of 8279 patients with primary hyperparathyroidism subjected to parathyroidectomy and 82,790 controls from the background population matched for age, gender and region

Characteristics	PHPT patients (*n* = 8279)		Control population (*n* = 82,790)	
Median age at index date, IQR	62 (53, 71)		62 (53, 71)	
*Age*	*n*	%	*n*	%
< 30	186	2.2	1860	2.2
30–39	337	4.1	3370	4.1
40–49	942	11.4	9420	11.4
50–59	1820	22.0	18,220	22.0
60–69	2451	29.6	24,510	29.6
70–79	1943	23.5	19,430	23.5
80+	598	7.2	5980	7.2
*Gender*				
Female	6374	77.0	63,740	77.0
Male	1905	23.0	19,050	23.0
*Education*	*n*	%	*n*	%
Elementary school	1972	23.8	20,643	24.9
Upper secondary school	3598	43.3	35,418	42.8
Post-secondary education	2612	31.6	25,347	30.6
Data not available	106	1.3	1382	1.7
*Occupational status*	*n*	%	*n*	%
Employed				
< 65 years	3385	75.6	34,949	78.1
65+ years	503	13.2	5008	13.2
Unemployed, declared				
< 65 years	248	5.5	2508	5.6
*Country of birth*				
Sweden	6897	83.1	69,063	83.4
Nordic countries (except Sweden)	445	5.4	3809	4.6
Europe (except Nordic countries)	475	5.7	5224	6.3
Outside Europé	480	5.8	5224	5.3
Unknown	0	–	2	–

*PHPT* primary hyperparathyroidism

**Table 2:** Preoperative characteristics in patients who underwent parathyroidectomy reported in The Scandinavian Quality Register of Thyroid, Parathyroid and Adrenal surgery (SQRTPA) in 2008 to 2013, *n* = 7177

	Female *n* = 5537		Male *n* = 1640	
	*n*	Median (25th; 75th)	*n*	Median (25th; 75th)
*Ionized calcium index date (mmol/L)*^a^				
< 50 years	973	1.44 (1.39; 1.50)	366	1.45 (1.41; 1.53)
50–64 years	2077	1.43 (1.38; 1.48)	552	1.45 (1.40; 1.51)
65–79 years	2092	1.43 (1.39; 1.49)	623	1.45 (1.40; 1.53)
80+ years	395	1.45 (1.40; 1.51)	99	1.51 (1.45; 1.60)
*Total calcium index date (mmol/L)*^b^				
All ages				
< 50 years	969	2.75 (2.66; 2.86)	362	2.77 (2.69; 2.91)
50–64 years	2061	2.73 (2.64; 2.82)	547	2.77 (2.67; 2.88)
65–79 years	2074	2.74 (2.65; 2.85)	613	2.77 (2.67; 2.91)
80+ years	393	2.77 (2.67; 2.90)	99	2.88 (2.77; 3.05)
*Ionized calcium 6 weeks postoperatively (mmol/L)*^a^				
< 50 years	888	1.24 (1.20; 1.27)	333	1.25 (1.21; 1.28)
50–64 years	1938	1.25 (1.21; 1.28)	506	1.23 (1.21; 1.27)
65–79 years	1949	1.24 (1.21; 1.28)	582	1.24 (1.20; 1.28)
80+ years	382	1.24 (1.21; 1.28)	86	1.22 (1.18; 1.26)
*Total calcium 6 months postoperatively (mmol/L)*^b^				
< 50 years	532	1.24 (1.21; 1.27)	202	1.24 (1.21; 1.28)
50–64 years	1172	1.24 (1.21; 1.27)	315	1.21 (1.23; 1.27)
65–79 years	1131	1.24 (1.21; 1.27)	327	1.19 (1.23; 1.26)
80+ years	230	1.23 (1.20; 1.28)	51	1.28 (1.23;1.26)
*Weight of excised parathyroid adenoma(s) (gram)*				
< 50 years	702	0.448 (0.255; 752)	284	0.499 (0.300; 794)
50–64 years	1557	0.400 (0.240; 657)	400	0.480 (0.280; 793)
65–79 years	1527	0.400 (0.250; 670)	423	0.600 (0.328; 0.900)
80+ years	291	0.479 (0.300; 0.750)	68	0.635 (0.432; 1.195)

Prevalent symptoms reported in The Scandinavian Quality Register of Thyroid, Parathyroid and Adrenal surgery (SQRTPA)										
	Kidney stone		Osteoporosis, osteopenia		Neuropsychiatric		Fatigue		None	
	*n*	%	*n*	%	*n*	%	*n*	%	*n*	%
All	448/7177	6.2	993/7177	13.8	756/7177	10.5	2172/7177	30.3	182/7177	2.5
< 50 years	117/1339	8.8	49/1339	3.6	115/1339	8.6	422/1339	31.5	44/1339	3.3
50–64 years	159/2629	6.0	328/2629	12.5	320/2629	12.2	819/2629	31.2	65/2629	2.5
65–79 years	155/2715	5.7	517/2715	19.0	261/2715	9.6	768/2715	28.3	61/2715	2.2
80+ years	17/494	3.4	99/494	20.0	60/494	12.1	163/494	33.0	12/494	2.2

*PTX* parathyroidectomy, *SQRTPA* The Scandinavian Quality Register of Thyroid, Parathyroid and Adrenal surgery

^a^Reference value ionized serum calcium; 1.15–1.33 mmol/L

^b^Reference value total plasma calcium; 2.15–2.50 mmol/L

**Fig. 2:**
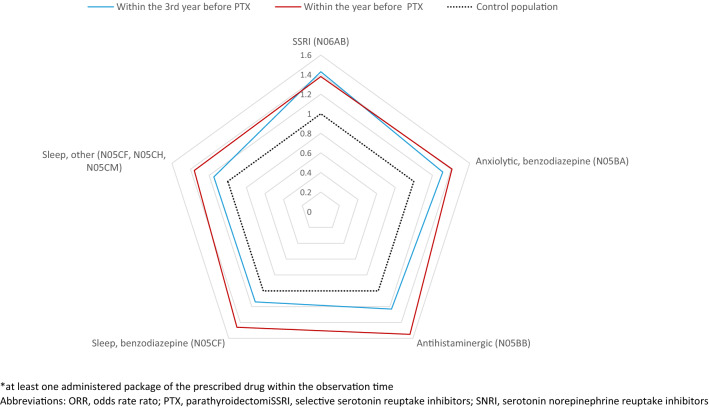
The most frequently used psychotropic drugs within three years before the index date among PHPT patents in relation to the control population (reference)*.

The retrospective analysis revealed that the utilization of psychotropic drugs was more extensive in patients prior to PTX than in the average population within 3 years prior to PTX (Table 3). Compared to the background population, the relative prevalence of treatment for sleep and anxiety in patients was greater the year prior to PTX as compared to a look back at the third year before index date (Fig. 2). The most common were benzodiazepines [OR 1.40 (95% CI 1.31–1.50)] and SSRI [1.38 (1.30–1.47)] for which ORs were highest among younger patients and patients with lower calcium levels; *p* < 0.001 (Table 3, Suppl.1). No significant gender-specific difference was observed. In the elderly (80 years of age and older), the utilization of antidepressive medication was similar in patients as compared to the background population, though there was increased use of benzodiazepines [OR 1.25 (95% CI 1.05–1.49)] in the PHPT cohort.

**Table 3:** Users by age groups and gender within 3 years prior to index date in the PHPT patients compared to the control population

				PHPT patients (*n* = 8279)		Control population (*n* = 82,790)		OR	95% CI
				*n*	%	*n*	%		
SSRI	N06AB	All patients		1316/8279	15.9	10,004/82,790	12.1	1.38	(1.30–1.47)
		Age	– 49	278/1465	19.0	1677/14,650	11.4	1.81	(1.57–2.09)
			50–64	516/3012	17.1	3627/30,120	12.0	1.53	(1.38–1.70)
			65–79	424/3204	13.2	3730/32,040	11.6	1.17	(1.05–1.30)
			80+	98/598	16.4	970/5980	16.2	0.98	(0.78–1.23)
		Sex	M	189/1905	9.9	1414/19,050	7.4	1.37	(1.17–1.61)
			F	1127/6374	17.7	8590/63,740	13.5	1.38	(1.29–1.48)
SNRI, other	N06AG, N06AX	All patients		715/8279	8.6	5396/82,790	6.5	1.36	(1.26–1.48)
		Age	– 49	132/1465	9.0	805/14,650	5.5	1.73	(1.42–2.10)
			50–64	292/3012	9.7	2099/30,120	7.0	1.45	(1.27–1.65)
			65–79	244/3204	7.6	1977/32,040	6.2	1.26	(1.09–1.44)
			80+	47/598	7.9	515/5980	8.6	0.90	(0.65–1.23)
		Sex	M	141/1905	7.4	888/19,050	4.7	1.65	(1.37–1.99)
			F	574/6374	9.0	4508/63,740	7.1	1.31	(1.19–1.43)
Tricyclic antidepressive	N06AA	All patients		374/8279	4.5	2723/82,790	3.3	1.40	(1.26–1.57)
		Age	– 49	58/1465	4.0	306/14,650	2.1	1.93	(1.44–2.58)
			50–64	169/3012	5.6	1083/30,120	3.6	1.61	(1.36–1.91)
			65–79	122/3204	3.8	1126/32,040	3.5	1.11	(0.91–1.34)
			80 +	25/598	4.2	208/5980	3.5	1.17	(0.76–1.81)
		Sex	M	47/1905	2.5	317/19,050	1.7	1.48	(1.08–2.03)
			F	327/6374	5.1	2406/63,740	3.8	1.39	(1.24–1.57)
Antihistaminergic	N05BB	All patients		716/8279	8.6	5023/82,790	6.1	1.47	(1.36–1.60)
		Age	– 49	171/1465	11.7	915/14,650	6.2	1.99	(1.67–2.38)
			50–64	281/3012	9.3	1943/30,120	6.5	1.51	(1.32–1.72)
			65–79	230/3204	7.2	1802/32,040	5.6	1.30	(1.13–1.51)
			80+	34/598	5.7	363/5980	6.1	0.93	(0.65–1.35)
		Sex	M	104/1905	5.5	749/19,050	3.9	1.42	(1.15–1.75)
			F	612/6374	9.6	4274/63,740	6.7	1.48	(1.36–1.62)
Anxiolytic, benzodiazepine	N05BA	All patients		1097/8279	13.3	8261/82,790	10.0	1.40	(1.31–1.50)
		Age	– 49	157/1465	10.7	800/14,650	5.5	2.09	(1.74–2.52)
			50–64	349/3012	11.6	2452/30,120	8.1	1.51	(1.33–1.70)
			65–79	464/3204	14.5	3784/32,040	11.8	1.28	(1.15–1.42)
			80+	127/598	21.2	1225/5980	20.5	1.06	(0.86–1.31)
		Sex	M	181/1905	9.5	1224/19,050	6.4	1.58	(1.34–1.87)
			F	916/6374	14.4	7037/63,740	11.0	1.37	(1.27–1.48)
Sleep, benzodiazepine	N05CF	All patients		1861/8279	22.5	14,589/82,790	17.6	1.38	(1.30–1.46)
		Age	– 49	208/1465	14.2	1229/14,650	8.4	1.82	(1.55–2.14)
			50–64	604/3012	20.1	4438/30,120	14.7	1.47	(1.34–1.62)
			65–79	816/3204	25.5	6909/32,040	21.6	1.25	(1.15–1.36)
			80+	233/598	39.0	2013/5980	33.7	1.25	(1.05–1.49)
		Sex	M	294/1905	15.4	2127/19,050	11.2	1.48	(1.29–1.70)
			F	1567/6374	24.6	12,462/63,740	19.6	1.36	(1.28–1.45)
Sleep, other	N05CF, N05CH, N05CM	All patients		667/8279	8.1	5032/82,790	6.1	1.36	(1.25–1.49)
		Age	– 49	120/1465	8.2	658/14,650	4.5	1.88	(1.53–2.31)
			50–64	278/3012	9.2	1873/30,120	6.2	1.54	(1.35–1.76)
			65–79	222/3204	6.9	2067/32,040	6.5	1.09	(0.95–1.26)
			80+	47/598	7.9	434/5980	7.3	1.10	(0.81–1.51)
		Sex	M	119/1905	6.2	915/19,050	4.8	1.35	(1.11–1.65)
			F	548/6374	8.6	4117/63,740	6.5	1.37	(1.25–1.50)
Neuroleptics	N05A, except Lithium			320/8279	3.9	2055/82,790	2.5	1.64	(1.45–1.85)
		Age	– 49	58/1465	4.0	290/14,650	2.0	2.09	(1.55–2.81)
			50–64	138/3012	4.6	693/30,120	2.3	2.13	(1.76–2.57)
			65–79	109/3204	3.4	827/32,040	2.6	1.40	(1.14–1.71)
			80+	15/598	2.5	245/5980	4.1	0.59	(0.34–1.02)
		Sex	M	73/1905	3.8	405/19,050	2.1	1.91	(1.47–2.47)
			F	247/6374	3.9	1650/63,740	2.6	1.57	(1.37–1.81)
Lithium	N05AN01			177/8279	2.1	319/82,790	0.4	5.74	(4.76–6.93)
		Age	– 49	19/1465	1.3	44/14,650	0.3	4.36	(2.49–7.62)
			50–64	89/3012	3.0	118/30,120	0.4	8.00	(6.04–10.59)
			65–79	66/3204	2.1	139/32,040	0.4	4.82	(3.58–6.49)
			80+	3/598	0.5	18/5980	0.3	1.72	(0.50–5.89)
		Sex	M	39/1905	2.0	56/19,050	0.3	7.11	(4.70–10.77)
			F	138/6374	2.2	263/63,740	0.4	5.44	(4.41–6.72)

At least one package administrated of the prescribed drug within the observation time

*PHPT* primary hyperparathyroidism, *SSRI* selective serotonin reuptake inhibitors, *SNRI* serotonin norepinephrine reuptake inhibitors

Both somatic and psychiatric diagnoses were more prevalent in the PHPT patient group. Psychiatric disorders (ICD-10; F06-F99) were more than twice as common before PTX with 8.7% among patients and 3.7% among the controls within the year prior to index date [OR 2.51 (CI 95% 1.31–2.73)]. In retrospect, the difference was less over a period of five years preoperatively [OR 1.67 (CI 95% 1.56–1.79)] (Table 4).

**Table 4:** Diagnoses registered in The National Patient Register (NPR) within one and five years prior to index date in patients compared to the control population

	PHPT cohort (*n* = 8279)		Control population (*n* = 82,790)		OR	95% CI
	*n*	%	*n*	%		
*Diagnoses ICD-10 registered within the period of one year prior to index date*						
Dementia, cognitive disorder, F00-F03	49	0.59	435	0.53	1.13	(0.84–1.52)
Psychiatric disorder, F06-F99	721	8.7	3032	3.7	2.51	(1.31–2.73)
Nervsystem, G00-G99	1549	18.7	11,583	13.0	1.42	(1.33–1.50)
Gastrointestinal disease, K20-K99	878	10.6	4493	5.4	2.07	(1.92–2.23)
Kidney stone, N20-N22	492	5.9	272	0.3	19.6	(16.5–22.26)
Musculoskeletal disease, M00-M79	1837	22.2	8684	10.5	1.43	(2.30–2.57)
*Diagnoses ICD-10 registered within the period of five years prior to index date*						
Dementia, cognitive disorder, F00-F03	58	0.7	855	1.3	0.68	(0.52–0.88)
Psychiatric disorder, F06-F99	1082	13.1	6821	8.4	1.67	(1.56–1.79)
Nervsystem, G00-G99	3206	38.7	27,018	32.6	1.30	(125–1.37)
Gastrointestinal disease, K20-K99	2052	24.8	13,254	16.0	1.73	(1.64–1.82)
Kidney stone, N20-N22	770	9.3	971	1.2	8.65	(7.84–9.53)
Musculoskeletal disease, M00-M79	3278	39.6	21,876	26.4	1.83	(1.74–1.91)

By the National Board of Health and Welfare

*PHPT* primary hyperparathyroidism, *ICD* international classification of disease

**Fig. 3:**
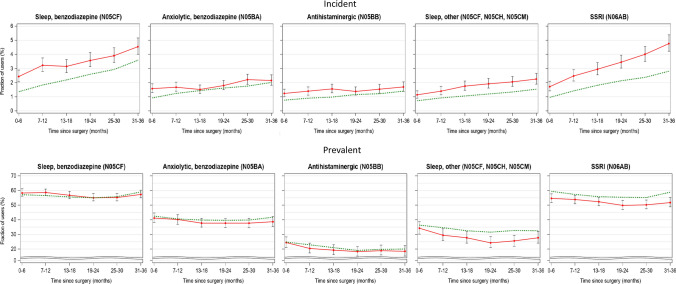
Proportion of PTX patients (red line) and population controls (green dashed line) filling prescriptions during three years after index date (i.e., date of surgery). Top panel shows incident users defined as subjects without filled prescriptions during the year before index date, and the bottom panel shows prevalent user defined as subjects with at least one filled prescription during the year before index date. Uncertainty intervals for the patients not intersecting the dashed green line indicates a significant (*p* < 0.05) difference in prescription rates between patients and controls

Figure 3 and Supplemental Table 2 illustrate initiation and continuation of drug use after PTX. Patients initiating treatment showed a significantly higher initiation rate after PTX than the controls for all substances during the first year after surgery, yet remained elevated at three years only for SSRI and benzodiazepine (sleep). Prevalent users showed the same prescription rate for patients and controls, with the exception of SSRI where patients show significantly lower usage than the controls.

Lithium treatment accounted for a relatively small proportion but was markedly over-represented in the patient cohort [OR 5.74 (CI 4.76–6.93)] which remained unaffected postoperatively (Table 3, Fig. 3, Suppl. Table 2).

## Discussion

This study aimed to map out the burden of mental illness in a population by means of analyzing data on the utilization of psychotropic drugs [22]. Thus far, the impact of PHPT on neuropsychiatric morbidity in relation to the background population has remained largely unknown. To our knowledge, this is the first large-scale population-based study of psychiatric comorbidity in PHPT patients prior to surgical treatment and the impact of PTX.

This analysis revealed that both the prevalence of psychiatric diagnoses and the utilization of psychotropic drugs were significantly more comprehensive in patients with PHPT before PTX as compared to the background population. The most commonly used drugs were SSRI, anxiolytic and sleep medication. Psychiatric comorbidity was found to be continuously increasing over at least three years of observation prior to PTX as compared to the background population indicating a progress of symptoms along with the duration of PHPT. The relative over-use in patients was found to be represented mainly in patients of up to 65 years of age. In the elderly, use of antidepressants was equivalent to the controls while sleep medication was slightly more common. However, neuropsychiatric symptoms were reported equally in the quality register SQRTPA irrespective of age. This may indicate that somatic symptoms tend to be more of a focal point in the elderly or in the restrictions on medication in order to await the effect of surgery [23].

Following PTX, prevalent users showed the same prescription rate for patients and controls, with the exception of SSRI where patients show a significantly lower prescription rate than the controls. In line with previous clinical studies, this supports the fact that curative PTX may have an effect on neuropsychiatric aspects of the disease even at a population level [4]. Contrarily, the initiation rates were higher for all substances up to one year postoperatively, yet remained significantly elevated at three years only for SSRI and benzodiazepine for sleep. However, numerically the initiation rates were low postoperatively; for SSRI 1.7% in patients and 1,0% in the controls and for benzodiazepines for sleep 2.4% and 1.3%, respectively, over six months after PTX.

Treatment with lithium, which is a known risk factor for developing PHPT, was found in 2.1% of the patients [24]. As expected, given that the main indication for lithium treatment is chronic psychiatric illness, medication with lithium was found to be stable both pre- and postoperatively. The causality of the observed excessive administration of psychotropic drugs in PHPT is elusive in this model. There are several hypothetical explanations for these results; e.g., symptoms of other origins may have prompted biochemical investigations that led to the diagnosis or prescribing of drugs may continue due to a lack of follow-ups with revision of medication [25]. Biochemical modulations due to the disease or to long-term symptomatic medication may further be subjects for explanatory hypotheses.

Although calcium is a well-known mediator in cell signaling throughout the nervous system, no convincing correlation between the level of hypercalcemia and neuropsychiatric symptoms in biochemically mild to moderate PHPT has been found [26]. The significant inverse correlation between excessive drug use and calcium levels found in this study is interesting. One possible explanation is that patients with psychiatric symptoms may have been prioritized for diagnostic work-up and treatment of PHPT.

The strength of this study is the large-scale population-based analysis of virtually comprehensive and valid data. The longitudinal study design allowed analysis of both the natural course of neuropsychiatric morbidity prior to PTX as well as the effects of treatment. In contrast to previous studies of self-reported QoL, this study comprehended objective registry-based data. The Swedish personal identification numbers enabled overlapping linkage between several well-validated national population registers, adding information on diagnoses, drug administrations and handling socioeconomic and demographic confounders [18, 27]. The measure of administered, prescribed drugs has previously been stated to constitute the best available proxy to estimate burden of disease in population-based studies and is considered more valid than diagnoses by ICD [22]. There are some arguments for this. For one, ICD codes tend to accumulate over time and inconsistent coding of diagnoses may lead to misclassification. In this setting, diagnose codes were not covered for primary care, which is of importance regarding conditions usually treated by general practitioners. In the Swedish health care system all psychotropic drugs are subsidized prescription-only, administered via pharmacies and thus completely covered in the drug register. Furthermore, the access to data from The Swedish Quality Register for Endocrine Surgery (SQRTPA) made it possible to include specific disease-related phenotypic characteristics in the analysis.

There are shortcomings in this study. Most importantly, the patients were already selected for treatment by various indications for surgery. Surveillance due to neuropsychiatric morbidity or other conditions are likely to infer selection bias. This aside, undiagnosed PHPT patients or patients treated conservatively were not captured by the data and this may have weakened the results. For these reasons, the generalizability is limited and the results in this study can only refer to patients already subjected to PTX. Moreover, data on the doses and the size of packages were not available for analyzing the magnitude of treatment. This was discussed. Based on the clinical fact that antidepressants, anxiolytics and sleep medication often are exchanged to a similar drug with different dosages or another brand, we considered that at least one allocation of a generic substance within 6 months as representative for estimating drug usage for continuous users. Nor can we say with certainty that the medication actually had been consumed. However, as this fact does not differ between patients and controls, the risk of affecting the result is minimal. Nevertheless, this new knowledge shines a light on the importance of penetrating the history of mental symptoms and taking them into account when assessing patients diagnosed with PHPT.

## Conclusion

PHPT is associated with increased medication for depression, anxiety and sleeping disorders. Prevalence of neuropsychiatric comorbidity should be assessed and considered in order to choose an optimal treatment for PHPT. Psychiatric status and the need for continuous medication should be reevaluated and followed up postoperatively.

## Supplementary Information

Below is the link to the electronic supplementary material.

Supplementary file1 (DOCX 739 kb)

Supplementary file2 (DOCX 703 kb)
