# Supplementary material for: Neuropsychiatric Comorbidity in Primary Hyperparathyroidism Before and After Parathyroidectomy: A Population Study
Source: World J Surg. 2022 Mar 5;46(6):1420–30. doi: 10.1007/s00268-022-06485-1 (PMC9054898; doi:10.1007/s00268-022-06485-1)
Supplement: Supplementary file 1 [file 268_2022_6485_MOESM1_ESM.docx]

Suppl. 1

	Ionized calcium (mmol/L)	PHPT patients (n=8279) n	%	Control population (n=82790) n	%	OR	95% CI	P-interaction
SSRI N06AB	**All patients**	1138/7141	15.9	1138/7141	12	1.39	(1.35-1.61)	
	**-1.38**	292/1598	18.3	292/1598	12.2	1.61	(1.39-1.99)	0.002
	**1.39-1.43**	310/1798	17.2	310/1798	12.2	1.52	(1.33-1.87)	
	1.44-1.49	299/1860	16.1	299/1860	12.5	1.36	(1.19-1.69)	
	1.50-	237/1885	12.6	237/1885	11.4	1.11	(1.02-1.51)	
SNRI, other N06AG, N06AX	**All patients**	622/7141	8.7	622/7141	6.5	1.39	(1.27-1.51)	
	**-1.38**	178/1598	11.1	178/1598	6.6	1.81	(1.53-2.14)	0.0013
	**1.39-1.43**	164/1798	9.1	164/1798	6.5	1.45	(1.22-1.72)	
	1.44-1.49	147/1860	7.9	147/1860	6.8	1.18	(0.98-1.41)	
	1.50-	133/1885	7.1	133/1885	6.1	1.18	(0.98-1.43)	
Tricyclic antidepressive N06AA	**All patients**	318/7141	4.5	318/7141	3.3	1.4	(1.24-1.58)	
	**-1.38**	87/1598	5.4	87/1598	3.2	1.71	(1.35-2.16)	0.1771
	**1.39-1.43**	85/1798	4.7	85/1798	3.4	1.46	(1.15-1.84)	
	1.44-1.49	76/1860	4.1	76/1860	3.5	1.19	(0.93-1.52)	
	1.50-	70/1885	3.7	70/1885	3	1.29	(0.99-1.67)	
Antihistaminergic N05BB	**All patients**	617/7141	8.6	617/7141	6.1	1.47	(1.35-1.61)	
	**-1.38**	157/1598	9.8	157/1598	6.2	1.67	(1.39-1.99)	0.134
	**1.39-1.43**	171/1798	9.5	171/1798	6.3	1.57	(1.33-1.87)	
	1.44-1.49	161/1860	8.7	161/1860	6.3	1.42	(1.19-1.69)	
	1.50-	128/1885	6.8	128/1885	5.6	1.24	(1.02-1.51)	
Anxiolytic, benzodiazepine N05BA	**All patients**	932/7141	13.1	932/7141	9.9	1.39	(1.29-1.50)	
	**-1.38**	247/1598	15.5	247/1598	9.9	1.72	(1.48-1.99)	0.004
	**1.39-1.43**	226/1798	12.6	226/1798	10	1.34	(1.15-1.55)	
	1.44-1.49	243/1860	13.1	243/1860	9.8	1.42	(1.22-1.64)	
	1.50-	216/1885	11.5	216/1885	10	1.17	(1.00-1.36)	
Sleep, benzodiazepine N05CF	**All patients**	1626/7141	22.8	1626/7141	17.7	1.4	(1.32-1.49)	
	**-1.38**	413/1598	25.8	413/1598	17.7	1.66	(1.47-1.88)	< 0.001
	**1.39-1.43**	445/1798	24.7	445/1798	18.3	1.51	(1.34-1.70)	
	1.44-1.49	395/1860	21.2	395/1860	17.5	1.3	(1.15-1.46)	
	1.50-	373/1885	19.8	373/1885	17.2	1.19	(1.05-1.35)	
Sleep, other N05CF, N05CH, N05CM	**All patients**	584/7141	8.2	584/7141	6.1	1.39	(1.29-1.50)	
	**-1.38**	142/1598	8.9	142/1598	6.3	1.47	(1.48-1.99)	0.7057
	**1.39-1.43**	151/1798	8.4	151/1798	6.1	1.43	(1.15-1.55)	
	1.44-1.49	155/1860	8.3	155/1860	6.2	1.39	(1.22-1.64)	
	1.50-	136/1885	7.2	136/1885	5.8	1.27	(1.00-1.36)	
Neuroleptics N05A, except Litium	**All patients**	281/7141	3.9	281/7141	2.5	1.69	(1.48-1.93)	
	**-1.38**	52/1598	3.3	52/1598	2.4	1.46	(1.09-1.96)	0.289
	**1.39-1.43**	63/1798	3.5	63/1798	2.5	1.52	(1.16-1.99)	
	1.44-1.49	75/1860	4	75/1860	2.5	1.73	(1.35-2.22)	
	1.50-	91/1885	4.8	91/1885	2.5	2.02	(1.59-2.57)	
**Lithium** N05AN01	**All patients**	152/7141	2.1	152/7141	0.4	5.58	(4.56-6.83)	
	**-1.38**	37/1598	2.3	37/1598	0.4	5.84	(3.88-8.79)	0.832
	**1.39-1.43**	34/1798	1.9	34/1798	0.4	4.71	(3.11-7.14)	
	1.44-1.49	36/1860	1.9	36/1860	0.4	5.79	(3.83-8.76)	
	1.50-	45/1885	2.4	45/1885	0.4	6.02	(4.13-8.77)	

*at least one package administrated of the prescribed drug within the observation time

Abbreviations: PHPT, primary hyperparathyroidism; SSRI, selective serotonin reuptake inhibitors; SNRI, serotonin norepinephrine reuptake inhibitors
