# Supplementary material for: Neuropsychiatric Comorbidity in Primary Hyperparathyroidism Before and After Parathyroidectomy: A Population Study
Source: World J Surg. 2022 Mar 5;46(6):1420–30. doi: 10.1007/s00268-022-06485-1 (PMC9054898; doi:10.1007/s00268-022-06485-1)
Supplement: Supplementary file 2 [file 268_2022_6485_MOESM2_ESM.docx]

Suppl. Table 2

SSRI (N06AB)	All users		Incident users**		Prevalent users***	
Time since index	RR	CI	RR	CI	RR	CI
0-6 months	1.25	(1.16-1.34)	1.8	(1.48-2.20)	0.92	(0.87-0.97)
7-12 months	1.28	(1.20-1.37)	1.75	(1.49-2.06)	0.94	(0.89-0.99)
13-18 months	1.27	(1.19-1.36)	1.62	(1.40-1.88)	0.94	(0.89-0.99)
19-24 months	1.25	(1.17-1.34)	1.62	(1.42-1.85)	0.9	(0.85-0.96)
25-30 months	1.28	(1.19-1.37)	1.68	(1.47-1.91)	0.91	(0.86-0.97)
31-36 months	1.27	(1.18-1.36)	1.69	(1.50-1.91)	0.88	(0.83-0.94)
P trend	*0.78*		*0.69*		*0.11*	

Anxiolytic, benzodiazepine (N05BA)	All users		Incident users**		Prevalent users***	
Time since index	RR	CI	RR	CI	RR	CI
0-6 months	1.31	(1.20-1.43)	1.72	(1.41-2.09)	0.97	(0.90-1.05)
7-12 months	1.28	(1.17-1.39)	1.37	(1.13-1.65)	0.99	(0.91-1.07)
13-18 months	1.17	(1.07-1.28)	1.05	(0.86-1.27)	0.95	(0.88-1.03)
19-24 months	1.18	(1.08-1.28)	1.1	(0.92-1.32)	0.95	(0.88-1.03)
25-30 months	1.21	(1.11-1.32)	1.25	(1.06-1.47)	0.95	(0.87-1.03)
31-36 months	1.14	(1.04-1.24)	1.07	(0.90-1.27)	0.93	(0.85-1.01)
P trend	*0.003*		*0.004*		*0.23*	

Sleep, benzodiazepine (N05CF)	All users		Incident users**		Prevalent users***	
Time since index	RR	CI	RR	CI	RR	CI
0-6 months	1.3	(1.23-1.38)	1.8	(1.52-2.14)	1.02	(0.98-1.07)
7-12 months	1.33	(1.25-1.41)	1.76	(1.52-2.05)	1.04	(1.00-1.08)
13-18 months	1.27	(1.19-1.34)	1.44	(1.24-1.66)	1.02	(0.98-1.07)
19-24 months	1.24	(1.17-1.32)	1.38	(1.21-1.59)	1	(0.96-1.05)
25-30 months	1.22	(1.15-1.29)	1.33	(1.17-1.52)	0.99	(0.95-1.04)
31-36 months	1.19	(1.12-1.26)	1.27	(1.12-1.44)	0.97	(0.93-1.02)
P trend	*<0001*		*0.004*		*0.005*	

Neuroleptics (N05A, except Lithium)	All users		Incident users**		Prevalent users***	
Time since index	RR	CI	RR	CI	RR	CI
0-6 months	1.69	(1.42-2.01)	2.04	(1.35-3.07)	0.98	(0.88-1.09)
7-12 months	1.65	(1.39-1.96)	1.6	(1.12-2.29)	0.98	(0.88-1.10)
13-18 months	1.57	(1.32-1.86)	1.28	(0.92-1.77)	0.97	(0.87-1.09)
19-24 months	1.59	(1.35-1.88)	1.27	(0.94-1.70)	1	(0.90-1.13)
25-30 months	1.46	(1.23-1.72)	1.02	(0.76-1.37)	0.98	(0.87-1.10)
31-36 months	1.45	(1.23-1.71)	1.02	(0.77-1.34)	1	(0.89-1.13)
P trend	*0.004*		*0.002.*		*0.66*	

Antihistaminergic (N05BB)	All users		Incident users**		Prevalent users***	
Time since index	RR	CI	RR	CI	RR	CI
0-6 months	**1.46**	**(1.28-1.66)**	**1.62**	**(1.31-2.01)**	**0.98**	**(0.85-1.14)**
7-12 months	**1.35**	**(1.18-1.55)**	**1.54**	**(1.25-1.89)**	**0.89**	**(0.75-1.05)**
13-18 months	**1.4**	**(1.23-1.6)**	**1.58**	**(1.30-1.92)**	**0.92**	**(0.77-1.09)**
19-24 months	**1.26**	**(1.10-1.45)**	**1.2**	**(0.98-1.47)**	**0.96**	**(0.80-1.14)**
25-30 months	**1.29**	**(1.13-1.48)**	**1.27**	**(1.04-1.54)**	**0.95**	**(0.80-1.14)**
31-36 months	**1.24**	**(1.07-1.43)**	**1.21**	**(0.99-1.47)**	**0.92**	**(0.76-1.12)**
P trend	*0.04*		*0.009*			

*at least one administered package of the prescribed drug within the periods of six months

**Incident users are defined as individual without a filled prescription of the drug the year before index date (i.e- date of surgery).

***Prevalent users are defined as individual with at least one filled prescription of the drug the year before index date (i.e- date of surgery).

Abbreviations: SSRI, selective serotonin reuptake inhibitors; PTX, parathyroidectomy patients; C, control population; m, months; RR, rate ratio; CI, confidence interval
